# Evaluation of Laboratory Management Based on a Combination of TOPSIS and RSR Methods: A Study in 7 Provincial Laboratories of China

**DOI:** 10.3389/fpubh.2022.883551

**Published:** 2022-07-11

**Authors:** Chihong Zhao, Bo Liu, Jing Li, Sisi Li, Yan Liu, Yuanyuan Guo, Xiumin Zhang

**Affiliations:** ^1^Department of Social Medicine and Health Management, School of Public Health, Jilin University, Changchun, China; ^2^Chinese Center for Disease Control and Prevention, Beijing, China

**Keywords:** laboratory management, evaluation, TOPSIS, RSR, combination methods

## Abstract

In this study, a comprehensive evaluation of management for pathogenic microbiology laboratories is performed based on a combination of Technique for Order Preference by Similarity to an Ideal Solution (TOPSIS) and Rank Sum Ratio (RSR) methods; in addition, the basis for improving laboratory management is provided. Using the laboratory evaluation tool developed by World Health Organization and a combination of TOPSIS and RSR methods, a system of evaluation indicators for the management of Chinese pathogenic microbiology laboratories is established for comprehensively evaluating the pathogenic microbiology laboratories of seven provincial Centers for Disease Control and Prevention. The evaluation system includes 12 primary indicators and 37 secondary indicators. In terms of laboratory management, the seven laboratories were ranked as D, G, E, C, F, B, and A in descending order. None of these laboratories were evaluated as “good” or “poor.” One of the laboratories was marked as “relatively poor” (A), two as “medium” (B and F), and four as “relatively good” (C, E, G, and D). In this study, a method for evaluating laboratory management using the TOPSIS and RSR methods is proposed, and a basis for comprehensively evaluating laboratory management for pathogenic microbiology laboratories is provided to reflect management practices.

## Introduction

Emerging and re-emerging infectious diseases remain a major threat to human health (1). Since the beginning of the 21st century, the world has experienced major crises caused by infectious diseases, including the emergence of severe acute respiratory syndrome in 2003 (2), H5N1 influenza in 1997 and its re-emergence in 2003 (3, 4), H1N1 in 2009 (5), Middle East respiratory syndrome in 2012 (6), Ebola virus disease in West Africa in 2014 (7, 8), and Coronavirus disease in 2019 (9).

Laboratories play an important role in detecting outbreaks of highly infectious diseases, risk assessment, early warning, early response and notification, and monitoring and surveillance (10, 11). Laboratory management determines a laboratory's detection capacity, which directly affects the effectiveness of the prevention and control of infectious diseases. Laboratory capacity has been a part of comprehensive planning of national and international public health response plans and has been a critical component of International Health Regulations (12).

Laboratory management has been widely studied, and many international organizations and countries have issued guidelines from different perspectives. World Health Organization (WHO) published the first edition of Laboratory Biosafety Manual in 1983; its fourth edition was published in 2020 (13). The latest version of ISO/IEC 17025 General requirements for the competence of testing and calibration laboratories was published in 2017, which can be used by laboratory customers, regulatory authorities, accreditation bodies, and others for confirming or recognizing the competence of laboratories (14). The sixth edition of the Biosafety in Microbiology and Biomedical Laboratories in USA (15), second edition of the Canadian Biosafety Standard (16), and others are good guides for laboratory management. In China, the national standard, General Requirements for Laboratory Biosafety (GB19489), stipulates the general requirements for the facilities, equipment, and safety management in laboratories with different biosafety levels (17). In addition, the national standard, General Requirements for the Competence of Testing and Calibration Laboratories (GB/T27025), specifies the general requirements pertaining to competence, fair practice, and consistent operation of laboratories (18).

Laboratory management includes a wide range of aspects, such as system construction, management system, data, personnel, equipment, testing capacity, and consumables management. A laboratory must improve the management level in an all-round fashion to achieve the desired capacity and role. Shortcomings in any aspect of laboratory management can directly affect the efficiency of the laboratory.

Standardized assessment is key to the development of comprehensive and integrated laboratory management. Inadequacies in laboratory management can be determined through evaluation; targeted capacity enhancement can be performed. WHO developed a laboratory assessment tool (LAT) in (19). The LAT describes a general process for the assessment of laboratories and provides questionnaires to help in the assessment of the national laboratory system and individual laboratories. Fourteen regional medical science centers have been evaluated using the LAT to enhance the public health laboratory capacity in Thailand (20).

Based on WHO's LAT, a comprehensive evaluation index system suitable for China's actual situation is proposed in the present study. Appropriate dimensions are determined, and indicators are prioritized to evaluate the performance of laboratory management in an appropriate model while considering local conditions.

The combination of Technique for Order Preference by Similarity to an Ideal Solution (TOPSIS) and Rank Sum Ratio (RSR) methods are mature comprehensive evaluation methods. These methods have been employed in different evaluation research (21–27). The TOPSIS method is a technique for order preference according to similarity to the ideal solution. Based on the normalized raw data matrix, this method forms a space for both positive and negative ideal solutions of priority solutions. The solutions to be evaluated are regarded as points in space. The distance between a point and the positive and ideal solutions is obtained. This distance helps identify the relative closeness between the solution to be evaluated and the positive ideal solution and provides a basis for evaluating the advantages and disadvantages of this solution (28, 29). The RSR method is a comprehensive evaluation and analysis method proposed by the Chinese statistician, Professor Fengtiao Tian, in 1988. The RSR method involves a matrix of n rows and m columns. The dimensionless statistic, RSR, is obtained through rank transformation. Statistical parameter analysis is used for studying the distribution of RSR. The RSR value is used to directly rank evaluation objects, rank evaluation objects by level, or compare the confidence intervals of the RSR for each group.

In the present study, a model is proposed for evaluating the performance of laboratory management and the performance of seven provincial Centers for Disease Control and Prevention (CDCs) laboratory management in China is analyzed using TOPSIS and RSR methods.

## Materials and Methods

### Subjects

Considering the regional economic development, local epidemics, as well as the prevention and control for infectious diseases, seven provincial CDCs—Guangxi, Guizhou, Yunnan, Hunan, Zhejiang, Guangdong, and Shanghai—represented by A, B, C, D, E, F, and G, respectively, were investigated.

### Evaluation Indicators

The evaluation indicators adopted herein are based on the LAT developed by WHO (19). The final system of comprehensive evaluation indicators for the management of pathogenic microbiology laboratory was devised through literature reviews, research and brainstorming, and experts' opinions and suggestions that reflect China's practices. This system includes 12 primary indicators, namely, organizational operation and management, documentation, sample collection, processing and transportation, data and information, consumables and reagents, equipment, analysis and testing capacities, quality control, facilities, human resources, biological risks and public health functions, as well as 37 secondary indicators of external and internal communication (Table 1).

**Table 1:** Proposed system of evaluation of comprehensive indicators for management of pathogenic microbiology laboratories.

**Primary indicators**	**Secondary indicators**
Organizational operation and management (χ1)	External communication (χ1.1) Internal communication (χ1.2) Funding guarantee (χ1.3) Laboratory qualification (χ1.4)
Documents (χ2)	Document management system (χ2.1) Quality management document (χ2.2) Biosafety management document (χ2.3)
Sample collection, processing and transportation (χ3)	Sample collection (χ3.1) Sample processing (χ3.2) Sample transportation (χ3.3)
Data and information (χ4)	Testing results and reports (χ4.1) Data analysis and statistics (χ4.2) Data security/confidentiality (χ4.3) Laboratory information management system (χ4.4)
Consumables and reagents (χ5)	Purchasing (χ5.1) Storage (χ5.2) Use (χ5.3) Management of expired reagent (χ5.4)
Equipment (χ6)	File management of equipment (χ6.1) Maintenance, calibration, and monitoring (χ6.2) Use and maintenance of key equipment (χ6.3)
Analysis and testing capacity (χ7)	Quantitative determination of bacteria (χ7.1) Quantitative determination of virus (χ7.2) Quantitative determination of parasites (χ7.3)
Quality control (χ8)	Internal quality control (IQC) (χ8.1) External quality control (EQA) (χ8.2) Review and evaluation (χ8.3)
Facilities (χ9)	Guarantee of working conditions (χ9.1)
Human resources (χ10)	Staff size that meets working requirements (χ10.1) Personnel qualifications and capabilities (χ10.2) Continuing Education (χ10.3)
Biological risks (χ11)	Documents of biological risk management (χ11.1) Biological risk assessment and control (χ11.2) Implementation and operation (χ11.3)
Public health functions (χ12)	Infectious disease monitoring and response (χ12.1) Sample exchange (χ12.2) Test reports (χ12.3)

### Data Collection and Quality Control

The questionnaires were filled by the CDCs as they sought information on a wide range of items and management of multiple departments. On-site reviews of the filled contents were performed by investigators. To ensure consistency in understanding and review, the team of investigators organized an intensive 2-day training courses for people who filled the questionnaires. During the process of filling in the questionnaires, telephone consultation service was provided. During on-site reviews, investigators conducted quality control of questionnaires through discussions and exchanges, document reviews, and laboratory inspections.

### Statistical Analysis

#### Weight Determination Method

Weight determination methods can be divided into two categories. (1) Subjective weighting, wherein the original data is primarily generated through experts' empirical judgments. It includes the direct evaluation method, Delphi method, analytic hierarchy process, and gray correlation method. (2) Objective weighting, wherein the actual data of each indicator in the evaluation process constitutes the original data. It comprises the coefficient of variation, principal component analysis, entropy method, and critic method. The two types of weighting methods have their own advantages and disadvantages. In subjective weighting methods, experts can reasonably determine the order of weight coefficients of various indicators to resolve actual problems, but with high subjectivity. In contrast, objective weighting methods are based on objective data but introduce an inevitable defect that the determined weights might contradict the actual importance of the indicators (30, 31).

Herein, the gray correlation method and the coefficient of variation method were employed as the subjective and objective weighting methods, respectively. The combined weight of the indicators was obtained by calculating the subjective and objective weights on certain coefficients.

#### Technique for Order Preference by Similarity to an Ideal Solution (TOPSIS)

Basic steps:

(1) The same trending method was used for all the indicators.

(2) The original data matrix with the same trend was normalized.


(1) $$Z_{ij}=\frac{x_{ij}}{\sqrt{\sum_{i=1}^{n}x_{ij}^{2}}}$$

In the equation, *x*_*ij*_ represents the value of the i-th evaluation object of the j-th indicator. The normalized matrix Z can be presented as


(2) $$\begin{array}{l} Z=\left[\begin{array}{cccc} Z_{11} & \;Z_{12} & \;\cdots & \;Z_{1m}\\ Z_{21} & \;Z_{22} & \;\cdots & \;Z_{2m}\\ \cdots & \;\cdots & \;\cdots & \;\cdots \\ Z_{n1} & \;Z_{n2} & \;\cdots & \;Z_{nm} \end{array}\right] \end{array}$$

(3) According to the normalized matrix Z, the positive ideal (optimal vector) and negative ideal solutions (worst-case vector) were calculated as follows:

Positive ideal solution: $Z^{+}=\left(z_{i1}^{+},z_{i2}^{+},\cdots \;,z_{im}^{+}\right)$.

Negative ideal solution: $Z^{-}=\left(z_{i1}^{-},z_{i2}^{-},\cdots \;,z_{im}^{-}\right)$.

In these equations, *i* = 1, 2,…, n and *j* = 1, 2, …, m. $Z_{ij}^{+}$ and $Z_{ij}^{-}$ respectively represent the maximum and minimum values of the evaluation object of the j-th indicator.

(4) The weighted Euclidean distance between $D_{i}^{+}$ (positive ideal solution) and $D_{i}^{-}$ (negative ideal solution) for each indicator values of every laboratory were calculated as follows:


(3) $$\begin{array}{lll} D_{i}^{+} & = & \sqrt{\overset{m}{\underset{j=1}{\sum }}{\left[\omega_{j}\left(Z_{ij}-Z_{ij}^{+}\right)\right]}^{2}} \end{array}$$

(4) $$\begin{array}{lll} D_{i}^{-} & = & \sqrt{\overset{m}{\underset{j=1}{\sum }}{\left[\omega_{j}\left(Z_{ij}-Z_{ij}^{-}\right)\right]}^{2}} \end{array}$$

In these equations, ω_*j*_ represents the weight coefficient of indicator *j*.

(5) The relative closeness between the positive ideal and negative ideal solutions as well as the indicator values for each laboratory were calculated as follows:


(5) $$\begin{array}{l} C_{i}=\frac{D_{i}^{-}}{D_{i}^{+}+D_{i}^{-}} \end{array}$$

The value of *C*_*i*_ was set between 0 and 1. A *C*_*i*_ value close to 1 indicates a higher likelihood of positive ideal solution (optimal level), whereas a *C*_*i*_ value close to 0 indicates a higher likelihood of negative ideal solution (worst level).

(6) Sorting of evaluation objects

To rank each evaluation object, the evaluation effect was determined by calculating the *C*_*i*_ value.

#### Rank Sum Ratio (RSR)

Basic steps:

(1) Data matrix

m evaluation indicators of n evaluation objects were arranged into a data table with n rows and m columns. The weight for each indicators was not equal; as such, the weight coefficients of the indicators were listed in different rows.

(2) Rank

The evaluation objects were ranked according to each evaluation indicator: the better the situation, the higher the rank.

(3) Calculation of RSR

In a matrix with n rows and m columns, the RSR was calculated as follows:


(6) $$\begin{array}{l} RSR_{i}=\frac{1}{m\cdot n}\overset{m}{\underset{j=1}{\sum }}R_{ij} \end{array}$$

In this equation, *i* = 1, 2, …, *n* and *j* = 1, 2, …, *m*. *R*_*ij*_ represents the rank of the indicator in the *i*-th row and *j*-th column.

In case the weight of each evaluation indicator was different, the weighted rank sum ratio was calculated as follows:


(7) $$\begin{array}{l} WRSR_{i}=\frac{1}{n}\overset{m}{\underset{j=1}{\sum }}W_{j}R_{ij} \end{array}$$

In this equation, *i* = 1, 2, …, *n* and *j* = 1, 2, …, *m*. *R*_*ij*_ represents the rank of indicator in the *i*-th row and *j*-th column, and *W*_*j*_ represents the weight of the *j*-th indicator, $\overset{m}{\underset{j=1}{\sum }}W_{j}=1$.

The RSR value was dimensionless. The minimum value was $RSR_{\text{min}}=\frac{1}{n}$, and the maximum value was *RSR*_max_ = 1.

(4) Determination of RSR distribution

The distribution of RSR refers to the specific downward cumulative frequency of RSR values expressed in the probability unit (Probit). The method includes the following steps:

① Compile the RSR frequency distribution table, list the frequency of each group *f*, and calculate the cumulative frequency ∑*f* of each group.

② Determine the rank *R* and average rank $\bar{R}$ of RSR in each group.

③ Calculate the downward cumulative frequency, $p=\bar{R}/n$.

④ Convert the percentage *p* to the Probit, which is the percentage *ps* corresponding to the standard normal deviation *u* plus 5.

(5) Calculation of the regression equation

Using the Probit corresponding to the cumulative frequency as the independent variable and RSR as the dependent variable, the regression equation was presented as *RŜR* = *a* + *b*× Probit.

(6) Rank by level

The evaluation objects were ranked by levels based on *RŜR* values.

#### Combination of TOPSIS and RSR Methods

The TOPSIS method can be applied to the relative closeness value *C*_*i*_, which ranges from 0 to 1, and the RSR value distributed in the same rank and ratio method can be analyzed using the RSR method.

Here are the basic steps involved:

(1) Determination of RSR distribution for Ci

The RSR distribution of Ci refers to the specific downward cumulative frequency of Ci expressed in the Probit. Specific steps include compiling the frequency distribution table of the Ci values; listing the frequency f of different groups and calculating the cumulative frequency ∑f of each group; determining the rank R, average rank R, and R/n values for each group of Ci values; refering to Appendix Table A1 to convert the percentage to the Probit; and obtaining the corresponding Probit value Y.

(2) Calculation of the regression equation

Using Y as the independent variable and Ci value as the dependent variable, the regression equation becomes Ci = a + b × Probit

(3) Hypothesis testing of the regression equation

Determine whether the Ci value is normally distributed and whether the regression equation is relevant.

(4) Rank by level

The evaluation objects are ranked by level based on the reasonable RSR rank method and the corresponding estimated values obtained using the regression equation.

Percentile and the corresponding Probit values at different levels are presented in Appendix Table A2. This study set 5 levels: poor, relatively poor, medium, relatively good, and good.

## Results

### Scores for Evaluation Indicators

Mean scores were calculated for the primary and secondary indicators. Results showed that χ11 (biological risks), χ8 (quality control), and χ12 (public health functions) scored the highest, with scores of 95.19, 94.05, and 91.27, respectively, while χ7 (analysis and testing capacity), χ4 (data and information), and χ1 (organizational operation and management) scored the lowest, with scores of 76.92, 83.39, and 85.63, respectively (Table 2).

**Table 2:** Scores for evaluation indicators.

**Primary indicators**	**Secondary indicators**	**A**	**B**	**C**	**D**	**E**	**F**	**G**	**Mean (secondary indicators)**	**Mean (primary indicators)**
χ1	χ1.1	78.13	83.33	96.88	100.00	100.00	100.00	100.00	94.05	85.63
	χ1.2	64.29	100.00	100.00	92.86	78.57	78.57	100.00	87.76	
	χ1.3	50.00	50.00	100.00	87.50	100.00	62.50	100.00	78.57	
	χ1.4	75.00	75.00	100.00	100.00	75.00	75.00	75.00	82.14	
χ2	χ2.1	75.00	83.33	83.33	83.33	83.33	83.33	83.33	82.14	90.14
	χ2.2	100.00	100.00	100.00	100.00	100.00	84.62	100.00	97.80	
	χ2.3	83.33	100.00	100.00	100.00	100.00	66.67	83.33	90.48	
χ3	χ3.1	83.33	99.48	100.00	97.30	97.40	81.25	100.00	94.11	87.22
	χ3.2	50.00	93.00	98.00	89.67	84.33	77.78	96.67	84.21	
	χ3.3	50.00	83.33	100.00	75.00	91.67	100.00	83.33	83.33	
χ4	χ4.1	72.38	100.00	100.00	97.96	84.62	96.70	100.00	93.09	83.39
	χ4.2	50.00	100.00	100.00	100.00	50.00	50.00	100.00	78.57	
	χ4.3	60.00	100.00	100.00	100.00	90.00	100.00	100.00	92.86	
	χ4.4	0.00	0.00	100.00	100.00	100.00	100.00	83.33	69.05	
χ5	χ5.1	62.50	87.50	87.50	87.50	87.50	87.50	100.00	85.71	87.19
	χ5.2	90.00	97.50	100.00	100.00	78.75	85.00	100.00	93.04	
	χ5.3	70.00	100.00	100.00	80.00	70.00	60.00	60.00	77.14	
	χ5.4	83.33	100.00	100.00	100.00	83.33	83.33	100.00	92.86	
χ6	χ6.1	83.33	100.00	100.00	100.00	98.33	96.67	83.33	94.52	85.76
	χ6.2	73.53	97.06	100.00	88.24	79.41	94.12	100.00	90.34	
	χ6.3	74.00	74.00	60.00	74.00	74.00	77.00	74.00	72.43	
χ7	χ7.1	83.33	94.44	94.44	94.44	94.44	83.33	83.33	89.68	76.92
	χ7.2	62.50	54.17	83.33	70.83	83.33	79.17	79.17	73.21	
	χ7.3	90.00	65.00	40.00	60.00	65.00	65.00	90.00	67.86	
χ8	χ8.1	90.00	91.67	100.00	100.00	91.67	75.00	100.00	92.62	94.05
	χ8.2	80.00	100.00	100.00	100.00	100.00	80.00	100.00	94.29	
	χ8.3	83.33	83.33	100.00	100.00	100.00	100.00	100.00	95.24	
χ9	χ9.1	45.00	95.00	85.00	95.00	95.00	94.44	100.00	87.06	87.06
χ10	χ10.1	50.00	48.98	100.00	90.82	91.84	48.98	100.00	75.80	87.06
	χ10.2	82.14	100.00	100.00	98.81	100.00	66.67	100.00	92.52	
	χ10.3	83.33	100.00	100.00	100.00	100.00	66.67	100.00	92.86	
χ11	χ11.1	100.00	100.00	100.00	100.00	100.00	66.67	100.00	95.24	95.19
	χ11.2	100.00	100.00	100.00	100.00	100.00	66.67	100.00	95.24	
	χ11.3	89.66	96.67	100.00	98.33	100.00	81.03	100.00	95.10	
χ12	χ12.1	83.33	100.00	100.00	100.00	100.00	66.67	100.00	92.86	91.27
	χ12.2	80.00	100.00	70.00	100.00	90.00	80.00	80.00	85.71	
	χ12.3	83.33	100.00	100.00	100.00	83.33	100.00	100.00	95.24	

### Weighting of Evaluation Indicators

The subjective weight, objective weight, and combination weight for a comprehensive indicator evaluation for pathogenic microbial laboratory management were obtained based on subjective weighting (gray relational analysis) and objective weighting (coefficient of variation). Results are summarized in Table 3. Three primary indicators had the highest weights: χ7 (analysis and testing capacity) (0.1067), χ1 (organizational operation and management) (0.1026), and χ12 (public health functions) (0.1024).

**Table 3:** Comprehensive evaluation of indicator weights for pathogenic microbial laboratory management.

**Primary indicators**	**Subjective weight**	**Objective weight**	**Weight combination**	**Secondary indicators**	**Subjective weight**	**Objective weight**	**Weight combination**
χ1	0.0441	0.0585	0.1026	χ1.1	0.0109	0.0092	0.0201
				χ1.2	0.0111	0.0114	0.0224
				χ1.3	0.0112	0.0094	0.0206
				χ1.4	0.0109	0.0286	0.0395
χ2	0.0423	0.0308	0.0731	χ2.1	0.0146	0.0126	0.0272
				χ2.2	0.0139	0.0090	0.0229
				χ2.3	0.0138	0.0092	0.0230
χ3	0.0431	0.0346	0.0777	χ3.1	0.0144	0.0033	0.0177
				χ3.2	0.0147	0.0083	0.0230
				χ3.3	0.0141	0.0229	0.0370
χ4	0.0407	0.0538	0.0945	χ4.1	0.0109	0.0049	0.0158
				χ4.2	0.0096	0.0138	0.0234
				χ4.3	0.0102	0.0096	0.0198
				χ4.4	0.0100	0.0254	0.0354
χ5	0.0385	0.0321	0.0706	χ5.1	0.0095	0.0059	0.0154
				χ5.2	0.0095	0.0096	0.0192
				χ5.3	0.0098	0.0109	0.0206
				χ5.4	0.0097	0.0056	0.0153
χ6	0.0397	0.0294	0.0691	χ6.1	0.0126	0.0028	0.0154
				χ6.2	0.0132	0.0055	0.0187
				χ6.3	0.0139	0.0211	0.0350
χ7	0.0411	0.0656	0.1067	χ7.1	0.0138	0.0187	0.0326
				χ7.2	0.0138	0.0214	0.0352
				χ7.3	0.0134	0.0256	0.0389
χ8	0.0414	0.0462	0.0876	χ8.1	0.0146	0.0115	0.0260
				χ8.2	0.0131	0.0160	0.0291
				χ8.3	0.0138	0.0187	0.0325
χ9	0.0403	0.0106	0.0509	χ9.1	0.0403	0.0106	0.0509
χ10	0.0440	0.0350	0.0790	χ10.1	0.0141	0.0211	0.0352
				χ10.2	0.0157	0.0031	0.0187
				χ10.3	0.0143	0.0109	0.0251
χ11	0.0453	0.0405	0.0857	χ11.1	0.0142	0.0161	0.0303
				χ11.2	0.0153	0.0170	0.0324
				χ11.3	0.0157	0.0073	0.0231
χ12	0.0395	0.0629	0.1024	χ12.1	0.0131	0.0241	0.0372
				χ12.2	0.0131	0.0208	0.0339
				χ12.3	0.0134	0.0179	0.0314

### Rank by Item

The seven provincial CDCs studied herein have their own strengths in terms of 12 aspects. The sub-item ranking of the primary indicators is summarized in Table 4.

**Table 4:** Sub-item ranking of the primary indicators for laboratory management.

**Indicator**		**A**	**B**	**C**	**D**	**E**	**F**	**G**
χ1	D^+^	0.0071	0.0062	0.0008	0.0008	0.0050	0.0057	0.0046
	D^−^	0.0009	0.0035	0.0070	0.0062	0.0045	0.0023	0.0055
	Ci	0.1084	0.3647	0.8966	0.8851	0.4732	0.2835	0.5452
	Ranking	7	5	1	2	4	6	3
χ2	D^+^	0.0019	0.0000	0.0000	0.0000	0.0000	0.0035	0.0016
	D^−^	0.0021	0.0036	0.0036	0.0036	0.0036	0.0010	0.0023
	Ci	0.5239	1.0000	1.0000	1.0000	1.0000	0.2319	0.5954
	Ranking	6	1	1	1	1	7	5
χ3	D^+^	0.0096	0.0028	0.0000	0.0042	0.0020	0.0024	0.0028
	D^−^	0.0001	0.0071	0.0097	0.0059	0.0078	0.0087	0.0074
	Ci	0.0151	0.7187	1.0000	0.5825	0.7988	0.7807	0.7284
	Ranking	7	5	1	6	2	3	4
χ4	D^+^	0.0176	0.0163	0.0000	0.0001	0.0055	0.0054	0.0027
	D^−^	0.0000	0.0065	0.0176	0.0176	0.0165	0.0167	0.0151
	Ci	0.0000	0.2842	1.0000	0.9926	0.7498	0.7568	0.8471
	Ranking	7	6	1	2	5	4	3
χ5	D^+^	0.0041	0.0009	0.0008	0.0022	0.0036	0.0043	0.0040
	D^−^	0.0013	0.0047	0.0047	0.0032	0.0020	0.0018	0.0032
	Ci	0.2432	0.8434	0.8485	0.6017	0.3488	0.2881	0.4466
	Ranking	7	2	1	3	5	6	4
χ6	D^+^	0.0024	0.0006	0.0031	0.0011	0.0017	0.0005	0.0012
	D^−^	0.0026	0.0033	0.0023	0.0030	0.0028	0.0036	0.0033
	Ci	0.5190	0.8479	0.4261	0.7363	0.6186	0.8772	0.7386
	Ranking	6	2	7	4	5	1	3
χ7	D^+^	0.0040	0.0074	0.0105	0.0067	0.0053	0.0055	0.0017
	D^−^	0.0106	0.0055	0.0055	0.0054	0.0076	0.0069	0.0115
	Ci	0.7246	0.4245	0.3415	0.4456	0.5903	0.5558	0.8709
	Ranking	2	6	7	5	3	4	1
χ8	D^+^	0.0033	0.0023	0.0000	0.0000	0.0009	0.0035	0.0000
	D^−^	0.0016	0.0029	0.0041	0.0041	0.0036	0.0021	0.0041
	Ci	0.3224	0.5571	1.0000	1.0000	0.8042	0.3786	1.0000
	Ranking	7	5	1	1	4	6	1
χ9	D^+^	0.0119	0.0011	0.0032	0.0011	0.0011	0.0012	0.0000
	D^−^	0.0000	0.0108	0.0087	0.0108	0.0108	0.0107	0.0119
	Ci	0.0000	0.9091	0.7273	0.9091	0.9091	0.8990	1.0000
	Ranking	7	2	6	2	2	5	1
χ10	D^+^	0.0087	0.0086	0.0000	0.0015	0.0014	0.0095	0.0000
	D^−^	0.0021	0.0042	0.0095	0.0082	0.0083	0.0000	0.0095
	Ci	0.1922	0.3299	1.0000	0.8409	0.8589	0.0000	1.0000
	Ranking	6	5	1	4	3	7	1
χ11	D^+^	0.0009	0.0003	0.0000	0.0002	0.0000	0.0061	0.0000
	D^−^	0.0059	0.0060	0.0061	0.0060	0.0061	0.0000	0.0061
	Ci	0.8612	0.9516	1.0000	0.9753	1.0000	0.0000	1.0000
	Ranking	6	5	1	4	1	7	1
χ12	D^+^	0.0044	0.0000	0.0045	0.0000	0.0025	0.0058	0.0030
	D^−^	0.0029	0.0070	0.0054	0.0070	0.0058	0.0025	0.0056
	Ci	0.3980	1.0000	0.5489	1.0000	0.6955	0.3045	0.6543
	Ranking	6	1	5	1	3	7	4

### Overall Ranking

Next, the 12 aspects were analyzed as a whole, and the seven provincial CDCs were ranked as D, G, E, C, F, B, and A (Table 5).

**Table 5:** Overall ranking of laboratory management.

	**A**	**B**	**C**	**D**	**E**	**F**	**G**
D^+^	0.0276	0.0217	0.0123	0.0085	0.0116	0.0180	0.0093
D^−^	0.0133	0.0202	0.0281	0.0274	0.0266	0.0235	0.0281
Ci	0.3246	0.4830	0.6951	0.7625	0.6968	0.5657	0.7503
Ranking	7	6	4	1	3	5	2

### Comprehensive Evaluation of RSR Distribution for Different Ci Values

*C*_*i*_ values were ranked in ascending order, and the downward cumulative frequency was calculated. According to the percentile value, refer to Appendix Table A1 and get the Y value. The corresponding Probit Y values are summarized in Table 6.

**Table 6:** Comprehensive evaluation of RSR distribution for different *C*_*i*_ values.

**CDC center**	***C*_*i*_**	**f**	**∑*f***	** $\bar{R}$ **	$\bar{R}/n(\%)$	**Y**
A	0.3246	1	1	1	14.29%	3.93
B	0.4830	1	2	2	28.57%	4.43
F	0.5657	1	3	3	42.86%	4.82
C	0.6951	1	4	4	57.14%	5.18
E	0.6968	1	5	5	71.43%	5.57
G	0.7503	1	6	6	85.71%	6.07
D	0.7625	1	7	7	96.43%^*^	6.80

* *Was calculated as 1 – 1/4n*.

Using the Probit value corresponding to the cumulative frequency as the independent variable and as the dependent variable, the regression equation was obtained as Ci = −0.1831 + 0.1511 × Y. At regression coefficient test statistic *t* = 5.0945, *P* < 0.05, indicating that the regression equation was relevant; at *F* = 25.954 and *P* < 0.05, the independent variable had a linear regression relationship with the dependent variable.

### Rank by Performance Level

The management of pathogenic microbiology laboratories in seven institutions was divided into five levels: poor, relatively poor, medium, relatively good, and good. None of the laboratories were marked as “poor” or “good”; one was marked as “relatively poor” (A); two were marked as “medium” (B and F); four were marked as “relatively good” (C, E, G, and D). See Table 7.

**Table 7:** Ranking of laboratory management evaluation in terms of performance.

	**Probit**	**Ci**	**Number of units**	**Composition %**	**Organization**
Poor	<3.2	0.3003	0	0.00%	
Relatively poor	3.2	0.3003	1	14.29%	A
Medium	4.4	0.4816	2	28.57%	B, F
Relatively good	5.6	0.6629	4	57.14%	C, E, G, D
Good	6.8	0.8441	0	0.00%	

## Discussion

Based on 12 primary indicators and 39 secondary indicators listed in the WHO's LAT, China's practices, literature reviews, and brainstorming, a comprehensive indicator evaluation system, including 12 primary indicators and 37 secondary indicators of management for pathogenic microbiology laboratories, was established herein. These indicators comprised organizational operation and management, documentation, sample collection, processing and transportation, data and information, consumables and reagents, equipment, analysis and testing capabilities, quality control, facilities, human resources, biological risks, and public health functions. Compared with the original LAT, the revised indicator system removed “gap analysis” from primary indicators and included “quality control.” Secondary indicators were adjusted under the framework of the new primary indicators. Although there were differences between the new evaluation system and LAT, the new one better reflects China's actual practices and meets the standard requirements. It also conforms to the WHO's concept of formulating LAT that encourages users to modify the LAT as per their own conditions. For example, a Thai study adopted 15 modules with quantitative output (20).

Management of laboratory biosafety risk has always been an important and difficult aspect of laboratory management. In this evaluation, χ11 (biological risks) had the highest score (95.19), which is largely explained by the continuous training provided by the Chinese disease control system. Training in biological risk management is critical, and should be ongoing conducted at all safety levels laboratories. However, χ7 (analysis and testing capacity) scored the lowest (76.92). Analysis revealed that the laboratories are efficient at detecting viruses and bacteria but are insufficient in parasite detection, which was responsible for overall low scores. With rapid economic development, greatly improved sanitary conditions, and a reduction in the need for parasite detection, laboratories have emphasized the development of bacterial and virus detection to ensure detection ability.

LAT does not give weight to each indicator, but the authors of this study believe that the role and impact of each indicator on laboratory management are different. We conclude that giving different weights to different indicators enables investigators to understand better the status and role of important indicators in the evaluation of the laboratory management process. However, as experts remain divided on the importance of each indicator, the weights assigned to various indicators vary from one expert to another. As such, in this study, both subjective and objective methods are employed for assigning weights to minimize the impact of subjective evaluation. The two types of weighting methods have their own advantages and disadvantages. The subjective weighting method entails experts to reasonably determine the rank of the weight coefficients of various indicators to resolve problems, resulting in a large extent of subjectivity. In contrast, the objective weighting method is based on objective data; however, the determined weights are, at times, contradictory to the actual importance of the indicators (28, 29). In the present study, the three primary indicators given the highest weights were χ7 (analysis and testing capacity), χ1 (organization operation and management), and χ12 (public health functions), whereas the three with the lowest weights were χ9 (facilities), χ6 (equipment), and χ5 (consumables and reagents). These results indicate that more attention should be paid to “analysis and testing capacity,” “organizational operation and management” and “public health functions” for the daily management of the laboratories.

Provincial CDCs play a crucial role in disease monitoring, prevention, and control, as well as public health decision-making. The level of laboratory management matters when it comes to the evaluation of CDC capacity building. In this study, we compared the differences in laboratory management of the provincial CDCs and identified areas in which the laboratories should strengthen the capacity building. Analyses revealed that laboratory A performed the poorest, with only χ7 (analysis and testing capacity) ranking top. B was number one in χ2 (documents), and χ12 (public health functions) but had low scores in χ4 (data and information) and χ7 (analysis and testing capacity), ranking sixth. C did well in 8 indicator, including χ1 (organizational operation and management), χ2 (documents) and so on, but lagged behind in χ6 (equipment) and χ7 (analysis and testing capacity). D had top scores in χ2 (documents), χ8 (quality control), and χ12 (public health functions), but ranked sixth in χ3 (sample collection, processing, and transportation). E ranked first in χ2 (documents) and χ11 (biological risks), with no indicators that were in the last place. F ranked first in χ6 (equipment) and last in χ2 (documents), χ10 (human resources), χ11 (biological risks), and χ12 (public health functions). G had the highest scores in χ7-χ11, with no last-ranked indicators. Therefore, this study will help CDC focus on the areas that need to be improved in comparison with other CDC and provides a reference for further efforts.

Pathogenic microbiology laboratories are not only built in the CDC system, but also widely exist in hospitals, scientific research institutes, universities, third-party medical institution, etc. The connotation and elements of laboratory management in different institutions are consistent. In this study, only 7 provincial CDCs were evaluated for laboratory management, hoping to provide some enlightenment for other institutions.

## Conclusions

Based on the LAT by WHO, an evaluation system for pathogenic microbiology laboratory was established in this study to comprehensively evaluate pathogenic microbiology laboratories in seven provincial CDCs by adopting a combination of TOPSIS and RSR methods. In the future, the evaluation system will be further optimized and promoted to devise a more objective evaluation for increased applicability to achieve scientific and accurate evaluation results, thus providing a basis for guiding laboratory capacity building and improving laboratory management.

## Data Availability Statement

The original contributions presented in the study are included in the article/Supplementary Material, further inquiries can be directed to the corresponding author.

## Author Contributions

Conceptualization: CZ, BL, and XZ. Data curation: CZ and BL. Formal analysis: CZ. Investigation: JL, SL, YL, and YG. Methodology, writing—original draft, writing—review, and editing: CZ and XZ. All authors read and approved the final manuscript.

## Funding

This research was funded by Public Health Emergency Response Mechanism Project of China CDC, grant number 21780404.

## Conflict of Interest

The authors declare that the research was conducted in the absence of any commercial or financial relationships that could be construed as a potential conflict of interest.

## Publisher's Note

All claims expressed in this article are solely those of the authors and do not necessarily represent those of their affiliated organizations, or those of the publisher, the editors and the reviewers. Any product that may be evaluated in this article, or claim that may be made by its manufacturer, is not guaranteed or endorsed by the publisher.

## Abbreviations

CDC: Centers for Disease Control and Prevention
LAT: Laboratory Assessment Tool
RSR: Rank Sum Ratio
TOPSIS: Technique for Order Preference by Similarity to an Ideal Solution
WHO: World Health Organization.
