# Supplementary material for: Evaluation of Laboratory Management Based on a Combination of TOPSIS and RSR Methods: A Study in 7 Provincial Laboratories of China
Source: Front Public Health. 2022 Jul 11;10:883551. doi: 10.3389/fpubh.2022.883551 (PMC9309487; doi:10.3389/fpubh.2022.883551)
Supplement: Supplementary file 1 [file Data_Sheet_1.DOC]

**Pathogenic Microbiology Laboratory Assessment Tool**

|  |
| --- |

| Lab Name： |  |
| --- | --- |
| Lab Address： |  |
| Lab Contact： | Telephone： |
| Evaluation Date： |  |
| Evaluator： |  |

| **1. Organizational operation and management** | | | | |
| --- | --- | --- | --- | --- |
| **NO.** | **Questions** | **Answers*** | **Comments** | |
|  | **External communication** |  |  | |
|  | Is the laboratory equipped with: |  | | |
| 1.1 | Telephone? |  |  | |
| 1.2 | Fax? |  |  | |
| 1.3 | Computer with Internet access? |  |  | |
| 1.4 | If yes or partial, does laboratory staff have access to the Internet? |  |  | |
| 1.5 | Is relevant information on costs and turnaround time for test results available? |  |  | |
| 1.6 | Is there timely notification to patients/customer when delay is anticipated due to machine breakdown, etc.? |  |  | |
| 1.7 | Do you organize customer surveys at least once a year? |  |  | |
|  | **Internal communication** |  |  | |
| 1.8 | Is there an organizational structure defining the lines of authorities and responsibilities for key laboratory staff? |  |  | |
| 1.9 | Are staff meetings organized at least once a month? |  |  | |
| 1.10 | If applicable, are team manager meetings organized at least once a month? |  |  | |
| 1.11 | Are meetings organized to solve a particular problem when it occurs? |  |  | |
| 1.12 | Are written reports or minutes of these meetings produced? |  |  | |
| 1.13 | Does laboratory staff participate in annual laboratory meetings specifically focused on sharing of knowledge and experiences and the opportunity to make improvements? |  |  | |
| 1.14 | Do laboratory representatives participate in institution board meetings as relevant? |  |  | |
|  | **Funding** |  |  | |
| 1.15 | Is fund sufficient for laboratory operation? |  |  | |
| 1.16 | Is the budget for staff salaries adequate for the need? |  |  | |
| 1.17 | Is there an adequate budget assigned for staff education? |  |  | |
| 1.18 | Is there an adequate budget assigned for consumable and reagent purchase? |  |  | |
| 1.19 | Is there an adequate budget assigned for equipment purchase/maintenance? |  |  | |
| 1.20 | *Please indicate the source of funds (proper funds, relevant ministry, NGO, specific networks, etc.)* |  |  | |
|  | **Laboratory qualification** |  |  | |
| 1.21 | Has the laboratory been licensed (i.e. authorized to operate) by the authorities? If yes or partial, please provide details |  |  | |
| 1.22 | Does the laboratory have an internal audit program? |  |  | |
| 1.23 | Has the laboratory undergone an audit or assessment by a third party (CNAS)? If yes or partial, please provide details |  |  | |
| 1.24 | Does the laboratory hold any form of certification (ISO 9001, other)? If yes or partial, please provide details |  |  | |
| **2. Documents** | | | | |
| **NO.** | **Questions** | **Answers** | **Comments** | |
|  | **Document management system** |  |  | |
| 2.1 | Is a system in place to organize the management of laboratory documents and records? |  |  | |
| 2.2 | Does the laboratory have a [procedure](javascript:;) for document management? |  |  | |
|  | If yes, are the documents: |  |  | |
| 2.3 | Listed? |  |  | |
| 2.4 | Numbered? |  |  | |
| 2.5 | Approved and signed by authorized personnel? |  |  | |
| 2.6 | Reviewed periodically? |  |  | |
| 2.7 | Archived? |  |  | |
| 2.8 | Does the laboratory have an archive system? |  |  | |
| 2.9 | Are the archived documents retrievable? |  |  | |
| 2.10 | *For how long are the archived documents kept?* |  |  | |
|  | **Quality management document** |  |  | |
| 2.11 | Is a quality manual describing the quality system policy and the quality procedures available? |  |  | |
|  | If yes or partial, does it cover these topics: |  | | |
| 2.12 | Laboratory organization and management? |  |  | |
| 2.13 | Documentation and records? |  |  | |
| 2.14 | Pre-examination procedures? |  |  | |
| 2.15 | Examination procedures? |  |  | |
| 2.16 | Post-examination procedures and reporting of results? |  |  | |
| 2.17 | Management of nonconformities? |  |  | |
| 2.18 | Personnel and education requirements? |  |  | |
| 2.19 | Safety and facilities? |  |  | |
| 2.20 | Equipment? |  |  | |
| 2.21 | Consumables and reagents? |  |  | |
| 2.22 | Reference materials? |  |  | |
| 2.23 | Collaboration with referral laboratories? |  |  | |
| 2.24 | Internal quality control procedures? |  |  | |
| 2.25 | External quality assessment procedures? |  |  | |
| 2.26 | Are all procedures related to relationship with other relevant institutes and organizations documented? |  |  | |
| 2.27 | Are procedures readily available to staff, as relevant? |  |  | |
| 2.28 | Are changes in procedures tracked? |  |  | |
| 2.29 | Are laboratory procedures reviewed at least annually and any necessary amendments incorporated? |  |  | |
| 2.30 | Are confidentiality protected and access limited, as appropriate? |  |  | |
| 2.31 | Are current versions of published standards and other similar documents in use in the laboratory available (e.g. norms, guidelines, instrument manuals, test kit inserts etc.)? |  |  | |
| 2.32 | Does the laboratory have procedures that were developed in-house? |  |  | |
| 2.33 | If so, are they clearly documented according to a defined format? |  |  | |
| 2.34 | Is there a procedure for the storage of primary samples once analyzed? |  |  | |
| 2.35 | Are there procedures for the validation and verification of methods and equipment as relevant? |  |  | |
| 2.36 | Are procedures in place to record incidents or complaints? |  |  | |
| 2.37 | If yes or partial, are corrective actions implemented and recorded? |  |  | |
|  | **Biosafety management document** |  |  | |
| 2.38 | Are written biosafety procedures available? |  |  | |
|  | If yes or partial, are the following subjects addressed: |  |  | |
| 2.39 | Personal protective equipment? |  |  | |
| 2.40 | Disinfection and sterilization? |  |  | |
| 2.41 | Waste disposal? |  |  | |
| 2.42 | Access restrictions? |  |  | |
| 2.43 | Biosafety equipment? |  |  | |
| 2.44 | Emergency protocols (e.g. in case of contamination)? |  |  | |
| 2.45 | Are Material Safety Data Sheets available for review in the immediate laboratory area? |  |  | |
| **3. Sample collection, procession and transportation** | | | | |
| **NO.** | **Questions** | **Answers** | **Comments** | |
|  | **Sample collection** |  |  | |
| 3.1 | Are written instructions available for patient preparation prior to collection (e.g. glucose tolerance test)? |  |  | |
| 3.2 | Are collection procedures documented and available to relevant personnel? |  |  | |
| 3.3 | Do these include minimum patient identification details? |  |  | |
| 3.4 | Is a standard sample request form available for those requesting tests? |  |  | |
|  | If yes or partial, does it include: |  |  | |
| 3.5 | Name of the patient? |  |  | |
| 3.6 | Gender? |  |  | |
| 3.7 | Date of birth? |  |  | |
| 3.8 | Patient identification number (if applicable)? |  |  | |
| 3.9 | Identification of the prescriber? |  |  | |
| 3.10 | Date of collection? |  |  | |
| 3.11 | Time of collection? |  |  | |
| 3.12 | Collector |  |  | |
| 3.13 | Type of Sample? |  |  | |
| 3.14 | Sample identification number (if applicable)? |  |  | |
| 3.15 | Examinations requested? |  |  | |
| 3.16 | Clinical information? |  |  | |
| 3.17 | Are Samples recorded in a book, worksheet, computer or other comparable system? |  |  | |
|  | If yes or partial, is there: |  |  | |
| 3.18 | A unique identification number? |  |  | |
| 3.19 | The date of receipt? |  |  | |
| 3.20 | The time of receipt? |  |  | |
| 3.21 | Receiver |  |  | |
| 3.22 | Are sample portions traceable to the original primary sample (identification number, etc.)? |  |  | |
|  | **Sample handling** |  |  | |
|  | Does the laboratory experience problems with samples from outside the facility due to (1.Never; 2.Sometimes; 3.Regularly; 4.Non applicable): | | | |
| 3.23 | No request form |  |  | |
| 3.24 | Incomplete request form |  |  | |
| 3.25 | Incorrect sample identification |  |  | |
| 3.26 | Incorrect patient identification |  |  | |
| 3.27 | Inadequate container |  |  | |
| 3.28 | Inadequate volume |  |  | |
| 3.29 | Inadequate transport media/anticoagulant |  |  | |
| 3.30 | Inadequate package |  |  | |
| 3.31 | Inadequate transportation temperature |  |  | |
| 3.32 | Delay in receipt |  |  | |
|  | Does the laboratory experience problems with collecting samples inside the facility due to (1.Never; 2.Sometimes; 3.Regularly; 4.Non applicable): | | | |
| 3.33 | Lack of proper collection materials |  |  | |
| 3.34 | No request form |  |  | |
| 3.35 | Incomplete request form |  |  | |
| 3.36 | Incorrect sample identification |  |  | |
| 3.37 | Incorrect patient identification |  |  | |
| 3.38 | Inadequate volume |  |  | |
| 3.39 | Others: |  |  | |
| 3.40 | Are there any criteria for acceptance or rejection of primary samples (including potential caution if non-conforming samples are accepted)? |  |  | |
| 3.41 | Are primary samples adequately stored if not immediately examined? |  |  | |
| 3.42 | Are samples stored for a specific time under appropriate conditions to enable further testing? |  |  | |
|  | **Sample referral / transport** |  |  | |
| 3.43 | Does the laboratory receive samples or isolates from other laboratories? (1.Never; 2.Sometimes; 3.Regularly; 4.Non applicable) |  |  | |
| 3.44 | Does the laboratory refer samples or isolates to other laboratories? (1.Never; 2.Sometimes; 3.Regularly; 4.Non applicable) |  |  | |
|  | *If yes or partial, please describe which samples to which laboratories in what circumstances:* |  |  | |
| 3.45 | Does the laboratory have appropriate packaging for referring samples? |  |  | |
| 3.46 | Is/are the person/s in charge of shipments trained for the transport of infectious substances? |  |  | |
|  | If yes or partial: |  |  | |
| 3.47 | Is he/she trained for local or national regulations or recommendations? |  |  | |
| 3.48 | Is he/she trained in international regulations? |  |  | |
| **4. Data and information** | | | | |
| **NO.** | **Questions** | **Answers** | **Comments** | |
|  | **Test results and reports** |  |  | |
| 4.1 | Are all original observations/results of the laboratory recorded in a worksheet or electronic database? |  |  | |
| 4.2 | Are results reported and recorded in a standardized format? |  |  | |
|  | If yes or partial, does the report form include the following: |  |  | |
| 4.3 | Name of the laboratory? |  |  | |
| 4.4 | Patient identification? |  |  | |
| 4.5 | Requester identification? |  |  | |
| 4.6 | Sample type? |  |  | |
| 4.7 | Examination method? |  |  | |
| 4.8 | Date of sample collection? |  |  | |
| 4.9 | Time of sample collection? |  |  | |
| 4.10 | Date of receipt of the sample by the laboratory? |  |  | |
| 4.11 | Time of receipt of the sample by the laboratory? |  |  | |
| 4.12 | Date of release of report? |  |  | |
| 4.13 | Time of release of report? |  |  | |
| 4.14 | Results reported in International System of Units (where applicable)? |  |  | |
| 4.15 | Biological reference intervals (where applicable)? |  |  | |
| 4.16 | Interpretation (where appropriate)? |  |  | |
| 4.17 | Identification and signature of the person authorizing the release of the report? |  |  | |
| 4.18 | Is there a system in place to track if reports have been issued and received? |  |  | |
| 4.19 | When samples need to be referred further to another laboratory, is there procedure to define how report is then issued and by which laboratory? |  |  | |
| 4.20 | Is there an immediate notification of physicians when results are critical for patient care? |  |  | |
| 4.21 | Is there an immediate notification of relevant ministry/surveillance network when results are critical? |  |  | |
|  | **Data analysis and statistics** |  |  | |
| 4.22 | Can the laboratory provide basic statistical data? |  |  | |
|  | **Data security - Confidentiality** |  |  | |
| 4.23 | Are access and modification of patient data protected for paper-based system? |  |  | |
| 4.24 | Are access and modification of patient data protected for electronic system? |  |  | |
| 4.25 | Is efficient back-up in place to prevent loss of patient result data in case of theft or other incident for paper-based system? |  |  | |
| 4.26 | Is efficient back-up in place to prevent loss of patient result data in case of theft or computer failure or other incident for electronic system? |  |  | |
| 4.27 | Are reported data (copies) retained as long as medically relevant or required by the legislation? |  |  | |
|  | **IT and Laboratory Information Management System (LIMS)** |  |  | |
| 4.28 | Is an electronic laboratory information management system used? |  |  | |
|  | If yes or partial: |  |  | |
| 4.29 | Are data retrievable within an acceptable timeframe? |  |  | |
| 4.30 | Can the system be used for data analysis? |  |  | |
| **5.** | **Consumables and reagents** |  |  | |
| **NO.** | **Questions** | **Answers** | **Comments** | |
|  | **Procurement** |  |  | |
| 5.1 | Are lists of manufacturers/suppliers and catalogues of reagents available? |  |  | |
| 5.2 | Does the laboratory experience problems with reagent delivery like delays, temperature not adequate, reference error, etc. (1.Never; 2.Sometimes; 3.Regularly; 4.Non applicable) |  |  | |
| 5.3 | Is there a responsible staff for consumable and reagent management (inventory, order, etc.)? |  |  | |
| 5.4 | Is the purchase of consumables and reagents recorded? |  |  | |
|  | **Inventory and storage** |  |  | |
| 5.5 | Is there an inventory system for consumables and reagents? |  |  | |
|  | If yes or partial, does it include: |  |  | |
| 5.6 | Quantity? |  |  | |
| 5.7 | Quality? |  |  | |
| 5.8 | Supplier? |  |  | |
| 5.9 | Lot number? |  |  | |
| 5.10 | Date of receipt? |  |  | |
| 5.11 | Appropriate storage? |  |  | |
| 5.12 | Expiration date? |  |  | |
| 5.13 | Date the material is placed in service? |  |  | |
| 5.14 | Are consumables and reagents inspected upon receipt? |  |  | |
| 5.15 | If yes or partial, are there protocols for acceptance/rejection of consumables and reagents? |  |  | |
| 5.16 | Are consumables and reagents appropriately stored (temperature, humidity, etc.)? |  |  | |
|  | **Use** |  |  | |
| 5.17 | Is the date of opening clearly written on the reagents/kits? |  |  | |
| 5.18 | Are new reagents (new product, new lot, including home-made reagents) validated against old reagents or reference materials before use? |  |  | |
| 5.19 | Is the consumption rate monitored for consumables and reagents? |  |  | |
| 5.20 | Is there a system for accurately forecasting needs for consumables and reagents? |  |  | |
| 5.21 | Are disposable supplies (e.g. tips, plastic pipettes, gloves) reused (1.Never; 2.Sometimes; 3.Regularly; 4.Non applicable)? |  |  | |
|  | **Expired reagents** |  |  | |
| 5.22 | Are expired reagents used (1.Never; 2.Sometimes; 3.Regularly; 4.Non applicable)? |  |  | |
| 5.23 | If sometimes or regularly, is quality control performed on these expired reagents? |  |  | |
| 5.24 | If sometimes or regularly, does quality control testing demonstrate that the quality of reagents is still acceptable? |  |  | |
| **6.** | **Equipment** |  |  | |
| **NO.** | **Questions** | **Answers** | **Comments** | |
|  | **Equipment inventory** |  |  | |
| 6.1 | Is there an equipment inventory? |  |  | |
| 6.2 | If yes or partial, is each equipment recorded with a paper or electronic equipment form? |  |  | |
|  | If yes or partial, does this form include: |  |  | |
| 6.3 | Name of the equipment? |  |  | |
| 6.4 | Serial number? |  |  | |
| 6.5 | Name and contact details of manufacturer (or local supplier)? |  |  | |
| 6.6 | Date of receipt? |  |  | |
| 6.7 | Date of first use? |  |  | |
| 6.8 | Location in the laboratory? |  |  | |
| 6.9 | Condition (i.e. new, used)? |  |  | |
| 6.10 | Maintenance activities? |  |  | |
| 6.11 | Calibration? |  |  | |
| 6.12 | The individual primarily responsible for this equipment? |  |  | |
|  | **Equipment maintenance, calibration and monitoring** |  |  | |
| 6.13 | Are results validated against reference materials and/or methods when new equipment is introduced? |  |  | |
| 6.14 | Is the equipment maintained in a safe working condition (including electrical safety)? |  |  | |
| 6.15 | Is there daily monitoring and recording of temperatures for temperature-dependent equipment? |  |  | |
| 6.16 | Is the staff duly trained and authorized before first using equipment? |  |  | |
| 6.17 | Do only authorized personnel use the equipment? |  |  | |
| 6.18 | Does the laboratory have a dedicated person in charge of the equipment (maintenance management, etc.)? |  |  | |
| 6.19 | Is a preventive maintenance program in place? |  |  | |
| 6.20 | Does the laboratory have contracts with external maintenance and repair services? |  |  | |
| 6.21 | Are data from equipment maintenance recorded and used? |  |  | |
| 6.22 | Are data from equipment use recorded? |  |  | |
| 6.23 | Is there a defined protocol and time period for pipette calibration? |  |  | |
| 6.24 | Is calibration of other equipment performed and checked regularly (pH meter, spectrophotometer, etc.)? |  |  | |
| 6.25 | Are results validated against reference materials after equipment maintenance or repair? |  |  | |
| 6.26 | Are there user manuals for most of the equipment? |  |  | |
| 6.27 | If yes, are these manuals available in the language commonly used by the staff? |  |  | |
| 6.28 | Are there sufficient spare parts for quick repairs (lamps, fuses, filters, etc.)? |  |  | |
| 6.29 | Is defective equipment (waiting for repair or obsolete to be removed) labeled appropriately? |  |  | |
|  | **Key Laboratory Equipment （Attached Table 1）** | | | |
| **7.** | **Analysis and testing performance** | | | |
| **NO.** | **Questions** | **Answers** | **Comments** | |
|  | **Pathogenic microbes the lab is able to test and identify** | | | |
|  | **Number of Pathogenic microbes (Attached Table 2)** |  |  | |
| 7.1 | Bacteria |  |  | |
| 7.2 | Virus |  |  | |
| 7.3 | Parasites |  |  | |
| **8. Quality Control** | | | | |
| **NO.** | **Questions** | **Answers** | **Comments** | |
|  | **Internal Quality Control (IQC)** |  |  | |
| 8.1 | Are there well-defined IQC protocol and procedures? |  |  | |
| 8.2 | Are standard materials used for IQC? |  |  | |
| 8.3 | Are drug resistance tests performed regularly? |  |  | |
| 8.4 | Is each batch of self-made culture media tested before use? |  |  | |
| 8.5 | Are self-made positive and negative controls used for each serological test? |  |  | |
| 8.6 | Are positive and negative controls used for each molecular test? |  |  | |
|  | **External Quality Assessment (EQA)** |  |  | |
| 8.7 | Did the lab participate in any EQA programs in the recent 3 years? |  |  | |
| 8.8 | If yes, were there any bacteriological programs? |  |  | |
| 8.9 | Were there any serological programs? |  |  | |
| 8.10 | Were there any molecular programs? |  |  | |
| 8.11 | Were there any virus isolation and culture programs? |  |  | |
|  | **Review and evaluation** |  |  | |
| 8.12 | Did the lab receive any external and internal assessment in the last year |  |  | |
| 8.13 | Is an internal assessment questionnaire established for laboratory quality assessment? |  |  | |
| 8.14 | Did the lab receive any advice and assistance from any other laboratory for laboratory quality assessment? |  |  | |
| **9.** | **Facilities** |  |  | |
| **NO.** | **Questions** | **Answers** | **Comments** | |
|  | **Guarantee of work conditions** |  |  | |
|  | What is the general condition of laboratory building and infrastructure? For the following questions, choose one of the following answers: 1.Good; 2.Medium; 3.Bad; 4.Non applicable |  |  | |
| 9.1 | Walls? |  |  | |
| 9.2 | Floors? |  |  | |
| 9.3 | Roofs? |  |  | |
| 9.4 | Windows and doors? |  |  | |
| 9.5 | Benches? |  |  | |
| 9.6 | Heating / air conditioner / ventilation? |  |  | |
| 9.7 | Lighting? |  |  | |
| 9.8 | Waste disposal? |  |  | |
| 9.9 | Liquid disposal system? |  |  | |
| 9.10 | Eye wash? |  |  | |
| 9.11 | Does the laboratory face electricity interruption (1.Never; 2.Sometimes; 3.Regularly; 4.Non applicable) |  |  | |
| 9.12 | If applicable, do you have an emergency electric generator or other backup power source? |  |  | |
| 9.13 | Is key/sensitive equipment protected by a UPS (Uninterruptable Power Supply)? |  |  | |
| 9.14 | Does the laboratory face water shortages (1.Never; 2.Sometimes; 3.Regularly; 4.Non applicable)? |  |  | |
| 9.15 | Is the space allocated sufficient to perform the work without compromising the quality and safety of patients and personnel? |  |  | |
| 9.16 | Are work areas clean and well maintained? |  |  | |
| 9.17 | Is sample collection carried out in room(s) separated from the laboratory examination room(s)? |  |  | |
| 9.18 | Is there an effective separation between adjacent laboratory sections in which there are incompatible activities (e.g. nucleic acid extraction vs. amplification)? |  |  | |
| 9.19 | Are there designated rooms for specialized testing (TB, brucellosis, etc.)? |  |  | |
| 9.20 | Are there appropriate storage areas? |  |  | |
| **10.** | **Human resources** |  | |  |
| **NO.** | **Questions** | **Answers** | **Comments** | |
|  | **Staff number** |  | |  |
| 10.1 | Is the staff number adequate to undertake the required work? |  | |  |
|  | If no or partial: |  | |  |
| 10.2 | Is trained manager/senior staff missing? |  | |  |
| 10.3 | Is technical staff missing? |  | |  |
| 10.4 | Is safety management staff missing? |  | |  |
| 10.5 | Is quality management staff missing? |  | |  |
| 10.6 | Is support/maintenance staff missing? |  | |  |
| 10.7 | Is laboratory disposal management staff missing? |  | |  |
| 10.8 | Is sample transport staff missing? |  | |  |
|  | **Qualifications** |  | |  |
| 10.9 | Are qualifications, training and experience of staff recorded? |  | |  |
| 10.10 | Are job descriptions defining qualifications and duties available? |  | |  |
| 10.11 | Are lines of authority and responsibility clearly defined for all laboratory staff? |  | |  |
| 10.12 | Has a quality manager been designated? |  | |  |
| 10.13 | Does the staff have appropriate qualifications or competences to perform laboratory work? |  | |  |
|  | If no or partial: |  | |  |
| 10.14 | Do manager/senior staff have appropriate qualifications or competences? |  | |  |
| 10.15 | Do laboratory technologists have appropriate qualifications or competences? |  | |  |
| 10.16 | Do safety/quality management staff have appropriate qualifications or competences? |  | |  |
| 10.17 | Do support/maintenance staff have appropriate qualificati**o**ns or competences? |  | |  |
| 10.18 | Do laboratory disposal management staff have appropriate qualifications or competences? |  | |  |
| 10.19 | Do other staff have appropriate qualifications or competences? |  | |  |
|  | **Continuous education** |  | |  |
| 10.20 | Is there a professional development program in place for the staff? |  | |  |
| 10.21 | Is continuing education (training, workshop, conference, etc.) provided to staff members? |  | |  |
| 10.22 | Is "in-house" education (on-site training, journal club, etc.) provided to staff members? |  | |  |
| **11.** | **Biorisk management** |  | |  |
| **NO.** | **Questions** | **Answers** | **Comments** | |
|  | **Biorisk management policy** |  | |  |
| 11.1 | Has a policy concerning the management of laboratory biorisk (biosafety and biosecurity) been written? |  | |  |
| 11.2 | Does this policy clearly state the biorisk management objectives and commitment to improve biorisk management performance? |  | |  |
| 11.3 | Is the policy appropriate to the nature and scale of the risk associated with the facility and associated activities? |  | |  |
|  | **Biorisk assessment and control** |  | |  |
| 11.4 | Have the biorisks been assessed and categorized? |  | |  |
| 11.5 | Are biorisk control measures described in an action plan? |  | |  |
| 11.6 | Are biorisk control measures documented? |  | |  |
|  | **Implementation and operation** |  | |  |
| 11.7 | Are roles and responsibilities related to biorisk management defined and documented? |  | |  |
| 11.8 | Is a senior manager designated to oversee the biorisk management system? |  | |  |
| 11.9 | Has a biorisk management committee been established? |  | |  |
| 11.10 | Is this management committee providing advice and guidance on biorisk management? |  | |  |
| 11.11 | Has this management committee delegated authority to stop work if necessary? |  | |  |
| 11.12 | Do personnel have access to occupational health services? |  | |  |
| 11.13 | Has a facility manager been designated to manage facilities, containment equipment and buildings? |  | |  |
| 11.14 | Has a security manager been designated? |  | |  |
| 11.15 | Has an animal care manager been designated, in case the laboratory handles animals? |  | |  |
| 11.16 | Are qualifications, experience and aptitudes relating to biorisk considered as part of the recruitment process? |  | |  |
| 11.17 | Is there mechanism/s to ensure that personnel are competent (e.g. successful completion of training, ability to perform tasks under supervision)? |  | |  |
| 11.18 | Is personnel regularly trained on biorisk management? |  | |  |
| 11.19 | Is an up-to-date biological agent and toxin inventory established and maintained? |  | |  |
| 11.20 | Are disinfection and decontamination procedures implemented effectively? |  | |  |
| 11.21 | Are waste management procedures implemented effectively? |  | |  |
| 11.22 | Are personal protective equipment and clothing used appropriately? |  | |  |
| 11.23 | Can personnel access prophylactic or emergency treatment in case of exposure to contaminated materials? |  | |  |
| 11.24 | Is a vaccination policy defined and implemented? |  | |  |
| 11.25 | Is behavior of personnel safe (e.g. no mouth pipetting, no recapping of needles, no smoking, no food stored in working areas)? |  | |  |
| 11.26 | Is there a formal commissioning process of new facilities? |  | |  |
| 11.27 | Are equipment and elements of the physical plant that may impact on biorisk identified? |  | |  |
| 11.28 | Are appropriate security measures in place to minimize potential inappropriate removal or release of biological agents (e.g. theft, earthquake, flood)? |  | |  |
| 11.29 | Is access to sensitive information (e.g. inventory of agents and toxins) controlled by adequate policies and procedures? |  | |  |
| 11.30 | Are procedures for a safe and secure transport of culture, samples and other contaminated materials established? |  | |  |
| 11.31 | Are emergency plans available (e.g. in case of explosion, fire, flood, worker exposure, accident or illness, major spillage)? |  | |  |
| 11.32 | Are emergency situation simulation exercises including security drills conducted at regular intervals? |  | |  |
| 11.33 | Are contingency measures planned in the event of an emergency or unforeseen event (e.g. power failure)? |  | |  |
| 11.34 | Are biorisk documents and records controlled and managed as part of the laboratory document management system? |  | |  |
| 11.35 | Are accident/incident and nonconformities related to biorisk correctly managed (i.e. reported, recorded, investigated, and leading to preventive or corrective actions)? |  | |  |
| 11.36 | Do planned inspection or audit/s include assessment of the biorisk management system? |  | |  |
| **12.** | **Public health functions** |  | |  |
| **NO.** | **Questions** | **Answers** | **Comments** | |
|  | **Surveillance and response** |  | |  |
| 12.1 | Is the laboratory part of surveillance network/s for endemic communicable diseases (e.g. HIV, parasitic diseases, hepatitis)? |  | |  |
| 12.2 | Has the laboratory defined responsibilities in national preparedness and response to public health emergencies like outbreaks? |  | |  |
| 12.3 | Are specific instructions or guidelines for laboratory investigation of public health events available? |  | |  |
|  | **Samples exchange** |  | |  |
| 12.4 | Does the laboratory receive samples from the field during the investigation of public health events or public health surveys? |  | |  |
| 12.5 | Does the laboratory give advice on sample collection and transport practices from the field during the investigation of public health emergencies? |  | |  |
| 12.6 | Does the laboratory have a stock of emergency laboratory sampling kits (personal protective equipment, sample collection material, transport media)? |  | |  |
| 12.7 | Is a procedure in place for the clinical laboratory to receive samples or isolates from public health laboratories for public health purpose? |  | |  |
| 12.8 | Does the clinical laboratory receive samples or isolates from public health laboratories for public health purpose? |  | |  |
| 12.9 | Is a procedure in place for the public health laboratory to receive samples or isolates from clinical laboratories for public health purpose (e.g. routine surveillance, outbreak investigation)? |  | |  |
| 12.10 | Does the public health laboratory receive samples or isolates from clinical laboratories for public health purpose (e.g. routine surveillance, outbreak investigation)? |  | |  |
| 12.11 | Does the laboratory refer samples or isolates to reference laboratories for public health purpose (e.g. routine surveillance, outbreak investigation)? |  | |  |
|  | **Test reports** |  | |  |
| 12.12 | Is a procedure in place to report notifiable diseases? |  | |  |
| 12.13 | Does the laboratory report notifiable diseases to the national disease surveillance network? |  | |  |
| 12.14 | Does the laboratory send aggregated data on a weekly or monthly basis to public health authorities? |  | |  |

**Possible answers (unless otherwise advised): 1.Yes; 2.Partial; 3.No; 4.Non applicable*

**Attached Table 1**

**Table 1 Key Laboratory Equipment**

| NO. | **Equipment List** | Number | Regular Maintenance? | Regular Calibration? |
| --- | --- | --- | --- | --- |
| 1 | Miniature automatic fluorescent enzyme label identification instrument |  |  |  |
| 2 | Radioimmunoassay |  |  |  |
| 3 | PCR Amplifier (real-time, fluorescence) |  |  |  |
| 4 | Ordinary PCR Amplifier |  |  |  |
| 5 | Microbial gene fingerprint identification system |  |  |  |
| 6 | Microplate reader |  |  |  |
| 7 | Multi-head pipette (set) |  |  |  |
| 8 | Air Microbial Sampler |  |  |  |
| 9 | Aquatic microbial membrane filtration device |  |  |  |
| 11 | Biosafety Cabinet |  |  |  |
| 17 | Microscope |  |  |  |
| 18 | Biological dissecting mirror |  |  |  |
| 19 | Inverted microscope |  |  |  |
| 20 | fluorescence microscope |  |  |  |
| 21 | electron microscope |  |  |  |
| 22 | Microtome |  |  |  |
| 23 | Nucleic acid protein transfer membrane instrument |  |  |  |
| 24 | hybridization furnace |  |  |  |
| 25 | Gel Imager |  |  |  |
| 26 | Viral load assay device |  |  |  |
| 27 | DNA sequencer |  |  |  |
| 28 | DNA shock meter |  |  |  |
| 29 | Chromatographic purification device |  |  |  |
| 30 | flow cytometer |  |  |  |
| 31 | Cryogenic high-speed centrifuge |  |  |  |
| 32 | High-precision constant temperature and humidity box |  |  |  |
| 33 | Mold incubator |  |  |  |
| 34 | CO2 incubator |  |  |  |
| 35 | Anaerobic incubator |  |  |  |
| 36 | Anaerobic Workstation |  |  |  |
| 37 | Tissue Section Production System |  |  |  |
| 38 | Cryostat (set) |  |  |  |
| 39 | liquid nitrogen tank |  |  |  |
| 40 | Program Cooler |  |  |  |
| 41 | Artificial climate box |  |  |  |
| 42 | PEET cabinet |  |  |  |
| 43 | Ultra Low Volume Sprayer |  |  |  |
| 44 | inhalation system |  |  |  |
| 45 | automatic blood cell counter |  |  |  |
| 46 | Ultrasonic cleaner |  |  |  |
| 47 | Ice maker |  |  |  |
| 48 | blood lactate meter |  |  |  |
| 49 | Multichannel Physiological Recorder |  |  |  |
| 50 | Urine analyzer |  |  |  |
| 51 | water maze |  |  |  |
| 52 | shuttle box |  |  |  |
| 53 | Laboratory Printers |  |  |  |
| 54 | Laboratory Computers |  |  |  |
| 55 | Vacuum Freeze Dryer |  |  |  |
| 56 | Automatic microbial identification and drug susceptibility testing system |  |  |  |
| 57 | Microbial Filtration Detection System |  |  |  |
| 58 | Horizontal nucleic acid electrophoresis instrument |  |  |  |
| 59 | Automatic plate washer |  |  |  |
| 60 | Ultra-clean workbench |  |  |  |
| 61 | dark field microscope |  |  |  |
| 62 | Ordinary centrifuge |  |  |  |
| 63 | Autoclave |  |  |  |
| 64 | Dry bake sterilizer |  |  |  |
| 65 | constant temperature incubator |  |  |  |
| 66 | Biochemical incubator |  |  |  |
| 67 | Constant temperature water bath |  |  |  |
| 68 | Air bath incubator shaker |  |  |  |
| 69 | Low temperature refrigerator (-20℃) |  |  |  |
| 70 | Low temperature refrigerator (-40℃) |  |  |  |
| 71 | Low temperature refrigerator (-70℃) |  |  |  |
| 72 | Low temperature refrigerator (-140℃) |  |  |  |
| 73 | Homogenizer |  |  |  |
| 74 | Micro shaker |  |  |  |
| 75 | Sample crusher |  |  |  |
| 76 | microwave digester |  |  |  |
| 77 | pure water processor |  |  |  |
| 78 | 1/10,000 electronic balance |  |  |  |
| 79 | 1/thousand electronic balance |  |  |  |
| 80 | Pulse Field Gel Electrophoresis Apparatus |  |  |  |
| 81 | UV/Visible Spectrophotometer |  |  |  |
| 82 | Office Printers |  |  |  |
| 83 | Office Computers |  |  |  |
| 84 | Semi-automatic strain identification or drug susceptibility testing system |  |  |  |
| 85 | refrigerator (4℃) |  |  |  |
| 86 | chemical fume hood |  |  |  |
| 87 | Thermostatic Magnetic Stirrer |  |  |  |
| 88 | PFGE analysis software |  |  |  |
| 89 | McFarlane Photometer |  |  |  |
| 90 | water bath shaker |  |  |  |
| 91 | pH meter |  |  |  |
| 92 | Automatic blood culture instrument |  |  |  |
| 93 | automatic hematology analyzer |  |  |  |
| 94 | vacuum pump |  |  |  |
| 95 | Turbidimeter |  |  |  |
| 96 | Automatic pipettes (not micropipettes) |  |  |  |

**Attached Table 2**

**Table 2 Analysis and testing performance of Pathogenic microbes**

|  | **Test Items** | **Evaluation** |
| --- | --- | --- |
| Bacteria | 1. Yersinia pestis |  |
|  | 2. Vibrio cholerae |  |
|  | 3. Bacillus anthracis |  |
|  | 4. Shigella dysentery |  |
|  | 5. Mycobacterium tuberculosis |  |
|  | 6. Typhoid fever, paratyphoid bacillus |  |
|  | 7. Bacillus pertussis |  |
|  | 8. Corynebacterium diphtheriae |  |
|  | 9. Tetanus bacillus |  |
|  | 10. Beta-hemolytic streptococcus (scarlet fever) |  |
|  | 11. Brucella |  |
|  | 12. Neisseria meningitidis |  |
|  | 13. Streptococcus suis |  |
|  | 14. Neisseria gonorrhoeae |  |
|  | 15. Mycobacterium leprae |  |
|  | 16. Escherichia coli O157:H7 |  |
|  | 17. Legionella pneumophila |  |
|  | 18. Salmonella |  |
| Virus | 1. SARS coronavirus |  |
|  | 2. HIV |  |
|  | 3. Hepatitis A virus |  |
|  | 4. HBV |  |
|  | 5. HCV |  |
|  | 6. Hepatitis D virus |  |
|  | 7. Hepatitis E virus |  |
|  | 8. Poliovirus |  |
|  | 9. Human infection with highly pathogenic avian influenza virus |  |
|  | 10. Epidemic hemorrhagic fever virus |  |
|  | 11. Rabies virus |  |
|  | 12. Japanese encephalitis virus |  |
|  | 13. Dengue virus |  |
|  | 14. Measles virus |  |
|  | 15. Influenza virus |  |
|  | 16. Mumps virus |  |
|  | 17. Rubella virus |  |
|  | 18. Rotavirus |  |
|  | 19. Monkeypox virus |  |
|  | 20. West Nile virus |  |
|  | 21. Nipah virus |  |
|  | 22. Sabia virus |  |
|  | 23. Ebola virus |  |
|  | 24. Bovine spongiform encephalovirus, prion (factor) |  |
| Parasites and Others | 1. Schistosoma |  |
|  | 2. Plasmodium |  |
|  | 3. Leishmania |  |
|  | 4. Filaria |  |
|  | 5. Toxoplasma gondii |  |
|  | 6. Ascaris |  |
|  | 7. Hookworms |  |
|  | 8. Clonorchis sinensis |  |
|  | 9. Cysticercosis |  |
|  | 10. Trichomonas vaginalis |  |
|  | 11. Angiostrongylus cantonii |  |
|  | 12. Mites |  |
|  | 13. Entamoeba histolytica |  |
|  | 14. Cryptosporidium |  |
|  | 15. Giardia |  |
|  | 16. Treponema pallidum |  |
|  | 17. Leptospira |  |
|  | 18. Rickettsia Platts |  |
|  | 19. Rickettsia Morsei |  |
|  | 20. Bartonella |  |
