# Supplementary material for: Evaluation of Laboratory Management Based on a Combination of TOPSIS and RSR Methods: A Study in 7 Provincial Laboratories of China
Source: Front Public Health. 2022 Jul 11;10:883551. doi: 10.3389/fpubh.2022.883551 (PMC9309487; doi:10.3389/fpubh.2022.883551)
Supplement: Supplementary file 2 [file Table_1.docx]

**Appendix A**

See Table A1.

Table A1 Percentile and the corresponding Probit.

| % | 0 | 0.1 | 0.2 | 0.3 | 0.4 | 0.5 | 0.6 | 0.7 | 0.8 | 0.9 |
| --- | --- | --- | --- | --- | --- | --- | --- | --- | --- | --- |
| 0 | – | 1.9098 | 2.1218 | 2.2522 | 2.3479 | 2.4242 | 2.4879 | 2.5427 | 2.5911 | 2.6344 |
| 1 | 2.6737 | 2.7096 | 2.7429 | 2.7738 | 2.8027 | 2.8299 | 2.8556 | 2.8799 | 2.9034 | 2.9251 |
| 2 | 2.9463 | 2.9665 | 2.9859 | 3.0046 | 3.0226 | 3.04 | 3.0569 | 3.0732 | 3.089 | 3.1043 |
| 3 | 3.1192 | 3.1337 | 3.1478 | 3.1616 | 3.1759 | 3.1881 | 3.2009 | 3.2134 | 3.2256 | 3.2376 |
| 4 | 3.2493 | 3.2608 | 3.2721 | 3.2831 | 3.294 | 3.3046 | 3.3151 | 3.3253 | 3.3354 | 3.3454 |
| 5 | 3.3551 | 3.3648 | 3.3742 | 3.3836 | 3.3928 | 3.4018 | 3.4107 | 3.4195 | 3.4282 | 3.4268 |
| 6 | 3.4452 | 3.4536 | 3.4618 | 3.4699 | 3.478 | 3.4859 | 3.4937 | 3.5015 | 3.5091 | 3.5167 |
| 7 | 3.5242 | 3.5316 | 3.5389 | 3.5462 | 3.5534 | 3.5606 | 3.5675 | 3.5745 | 3.5813 | 3.5882 |
| 8 | 3.5949 | 3.6016 | 3.6083 | 3.6148 | 3.6213 | 3.6278 | 3.6342 | 3.6405 | 3.6468 | 3.6531 |
| 9 | 3.6592 | 3.6654 | 3.6715 | 3.6775 | 3.6835 | 3.6894 | 3.6953 | 3.7012 | 3.707 | 3.7127 |
| 10 | 3.7184 | 3.7241 | 3.7298 | 3.7354 | 3.7409 | 3.7464 | 3.7519 | 3.7547 | 3.7625 | 3.7681 |
| 11 | 3.7735 | 3.7788 | 3.784 | 3.7893 | 3.7945 | 3.7996 | 3.8048 | 3.8099 | 3.815 | 3.82 |
| 12 | 3.825 | 3.83 | 3.835 | 3.8399 | 3.8448 | 3.8497 | 3.8545 | 3.8593 | 3.8641 | 3.8689 |
| 13 | 3.8736 | 3.8783 | 3.883 | 3.8877 | 3.8923 | 3.8969 | 3.9015 | 3.9061 | 3.9107 | 3.9152 |
| 14 | 3.9197 | 3.9242 | 3.9268 | 3.9331 | 3.9375 | 3.9419 | 3.9463 | 3.9506 | 3.955 | 3.9593 |
| 15 | 3.9636 | 3.9678 | 3.9721 | 3.9763 | 3.9806 | 3.9848 | 3.989 | 3.9931 | 3.9973 | 4.0014 |
| 16 | 4.0055 | 4.0096 | 4.0137 | 4.0178 | 4.0218 | 4.0259 | 4.0299 | 4.0339 | 4.0379 | 4.0419 |
| 17 | 4.0458 | 4.0498 | 4.0537 | 4.0576 | 4.0615 | 4.0654 | 4.0693 | 4.0731 | 4.077 | 4.0808 |
| 18 | 4.0846 | 4.0884 | 4.0922 | 4.096 | 4.0998 | 4.1035 | 4.1073 | 4.111 | 4.1147 | 4.1184 |
| 19 | 4.1221 | 4.1258 | 4.1295 | 4.1331 | 4.1367 | 4.1404 | 4.144 | 4.1476 | 4.1512 | 4.1548 |
| 20 | 4.1584 | 4.1619 | 4.1655 | 4.169 | 4.1726 | 4.1761 | 4.1796 | 4.1831 | 4.1866 | 4.1901 |
| 21 | 4.1936 | 4.197 | 4.2005 | 4.2039 | 4.2074 | 4.2108 | 4.2142 | 4.2176 | 4.221 | 4.2244 |
| 22 | 4.2278 | 4.2312 | 4.2345 | 4.2379 | 4.2412 | 4.2446 | 4.2479 | 4.2512 | 4.2546 | 4.2579 |
| 23 | 4.2612 | 4.2644 | 4.2677 | 4.271 | 4.2743 | 4.2775 | 4.2808 | 4.284 | 4.2872 | 4.2905 |
| 24 | 4.2937 | 4.2969 | 4.3001 | 4.3033 | 4.3065 | 4.3097 | 4.3129 | 4.316 | 4.3192 | 4.3224 |
| 25 | 4.3255 | 4.3287 | 4.3318 | 4.3349 | 4.338 | 4.3412 | 4.3443 | 4.3474 | 4.3505 | 4.3536 |
| 26 | 4.3567 | 4.3597 | 4.3628 | 4.3659 | 4.3689 | 4.372 | 4.375 | 4.3781 | 4.3811 | 4.3842 |
| 27 | 4.3872 | 4.3908 | 4.3932 | 4.3962 | 4.3992 | 4.4022 | 4.4052 | 4.4082 | 4.4112 | 4.4142 |
| 28 | 4.4172 | 4.4201 | 4.4231 | 4.426 | 4.429 | 4.4319 | 4.4349 | 4.4378 | 4.4408 | 4.4437 |
| 29 | 4.4466 | 4.4495 | 4.4524 | 4.4554 | 4.4583 | 4.4612 | 4.4641 | 4.467 | 4.4698 | 4.4727 |
| 30 | 4.4756 | 4.4785 | 4.4813 | 4.4842 | 4.4871 | 4.4899 | 4.4982 | 4.4956 | 4.4985 | 4.5013 |
| 31 | 4.5041 | 4.505 | 4.5098 | 4.5129 | 4.5155 | 4.5183 | 4.5211 | 4.5239 | 4.5267 | 4.5295 |
| 32 | 4.5323 | 4.5351 | 4.5379 | 4.5407 | 4.5435 | 4.5462 | 4.549 | 4.5518 | 4.5546 | 4.5573 |
| 33 | 4.5601 | 4.5628 | 4.5656 | 4.5684 | 4.5711 | 4.5739 | 4.5766 | 4.5793 | 4.5821 | 4.5845 |
| 34 | 4.5875 | 4.5903 | 4.593 | 4.5957 | 4.5984 | 4.6011 | 4.6039 | 4.6066 | 4.6093 | 4.612 |
| 35 | 4.6147 | 4.6174 | 4.6201 | 4.6228 | 4.6255 | 4.6281 | 4.6308 | 4.6335 | 4.6362 | 4.6389 |
| 36 | 4.6415 | 4.6442 | 4.6469 | 4.6495 | 4.6522 | 4.6549 | 4.6575 | 4.6602 | 4.6628 | 4.6655 |
| 31 | 4.6681 | 4.6708 | 4.6734 | 4.6761 | 4.6787 | 4.6814 | 4.684 | 4.6866 | 4.6893 | 4.6919 |
| 38 | 4.6945 | 4.6971 | 4.6992 | 4.7024 | 4.705 | 4.7076 | 4.7102 | 4.7129 | 4.7155 | 4.7181 |
| 39 | 4.7207 | 4.7233 | 4.7259 | 4.7285 | 4.7311 | 4.7337 | 4.7363 | 4.7389 | 4.7415 | 4.7441 |
| 40 | 4.7467 | 4.7492 | 4.7518 | 4.7544 | 4.757 | 4.7596 | 4.7622 | 4.7647 | 4.7673 | 4.7699 |
| 41 | 4.7725 | 4.775 | 4.7776 | 4.7802 | 4.7827 | 4.7853 | 4.7879 | 4.7904 | 4.793 | 4.7955 |
| 42 | 4.7981 | 4.8007 | 4.8032 | 4.8058 | 4.8083 | 4.8109 | 4.8134 | 4.816 | 4.8185 | 4.8211 |
| 43 | 4.8236 | 4.8262 | 4.8287 | 4.8313 | 4.8338 | 4.8363 | 4.8389 | 4.8414 | 4.844 | 4.8465 |
| 44 | 4.849 | 4.8516 | 4.8541 | 4.8566 | 4.8592 | 4.8617 | 4.8642 | 4.8668 | 4.8693 | 4.8718 |
| 45 | 4.8743 | 4.8769 | 4.8794 | 4.8819 | 4.8844 | 4.887 | 4.8895 | 4.892 | 4.8945 | 4.897 |
| 46 | 4.8995 | 4.9021 | 4.9046 | 4.9071 | 4.9096 | 4.9122 | 4.9147 | 4.9172 | 4.9197 | 4.9222 |
| 47 | 4.9247 | 4.9272 | 4.9298 | 4.9323 | 4.9358 | 4.9373 | 4.9398 | 4.9423 | 4.9448 | 4.9473 |
| 48 | 4.9498 | 4.9524 | 4.9549 | 4.9574 | 4.9599 | 4.9624 | 4.9649 | 4.9674 | 4.9699 | 4.9724 |
| 49 | 4.9749 | 4.9774 | 4.9799 | 4.9825 | 4.985 | 4.9875 | 4.99 | 4.9925 | 4.995 | 4.9975 |
| 50 | 5 | 5.0025 | 5.005 | 5.0075 | 5.01 | 5.0125 | 5.015 | 5.0175 | 5.0201 | 5.0226 |
| 51 | 5.0251 | 5.0276 | 5.0301 | 5.0326 | 5.0351 | 5.0376 | 5.0401 | 5.0426 | 5.0451 | 5.0476 |
| 52 | 5.0502 | 5.0527 | 5.0552 | 5.0577 | 5.0602 | 5.0627 | 5.0652 | 5.0677 | 5.0702 | 5.0728 |
| 53 | 5.0753 | 5.0778 | 5.0803 | 5.0828 | 5.0853 | 5.0878 | 5.0904 | 5.0929 | 5.0954 | 5.0979 |
| 54 | 5.1004 | 5.103 | 5.1055 | 5.108 | 5.1105 | 5.113 | 5.1156 | 5.1181 | 5.1206 | 5.1231 |
| 55 | 5.1257 | 5.1282 | 5.1307 | 5.1332 | 5.1358 | 5.1383 | 5.1408 | 5.1434 | 5.1459 | 5.1484 |
| 56 | 5.151 | 5.1535 | 5.156 | 5.1586 | 5.1611 | 5.1637 | 5.1662 | 5.1687 | 5.1713 | 5.1738 |
| 57 | 5.1764 | 5.1789 | 5.1815 | 5.184 | 5.1866 | 5.1891 | 5.1917 | 5.1942 | 5.1968 | 5.1993 |
| 58 | 5.2019 | 5.2045 | 5.207 | 5.2096 | 5.2121 | 5.2147 | 5.2173 | 5.2198 | 5.2224 | 5.225 |
| 59 | 5.2275 | 5.2301 | 5.2327 | 5.2353 | 5.2378 | 5.2404 | 5.243 | 5.2456 | 5.2482 | 5.2508 |
| 60 | 5.2533 | 5.2559 | 5.2585 | 5.2611 | 5.2627 | 5.2663 | 5.2689 | 5.2715 | 5.2741 | 5.2767 |
| 61 | 5.2793 | 5.2819 | 5.2845 | 5.2871 | 5.2898 | 5.2924 | 5.295 | 5.2976 | 5.3002 | 5.3029 |
| 62 | 5.3055 | 5.3081 | 5.3107 | 5.3134 | 5.316 | 5.3186 | 5.3213 | 5.3239 | 5.3266 | 5.3292 |
| 63 | 5.3319 | 5.3345 | 5.3372 | 5.3398 | 5.3425 | 5.3451 | 5.3478 | 5.3505 | 5.3531 | 5.3558 |
| 64 | 5.3585 | 5.3611 | 5.3638 | 5.3665 | 5.3692 | 5.3719 | 5.3745 | 5.3772 | 5.3799 | 5.3826 |
| 65 | 5.3853 | 5.388 | 5.3907 | 5.3934 | 5.3961 | 5.3989 | 5.4016 | 5.4043 | 5.407 | 5.4097 |
| 66 | 5.4125 | 5.4152 | 5.4179 | 5.4207 | 5.4234 | 5.4261 | 5.4289 | 5.431 | 5.4344 | 5.4372 |
| 67 | 5.4399 | 5.4427 | 5.4454 | 5.4482 | 5.451 | 5.4538 | 5.4565 | 5.4593 | 5.4621 | 5.4649 |
| 68 | 5.4677 | 5.4705 | 5.4733 | 5.4761 | 5.4689 | 5.4817 | 5.4845 | 5.4874 | 5.4902 | 5.493 |
| 69 | 5.4858 | 5.4987 | 5.5015 | 5.5044 | 5.5072 | 5.5101 | 5.5129 | 5.5158 | 5.5187 | 5.5215 |
| 70 | 5.5244 | 5.5273 | 5.5302 | 5.533 | 5.5359 | 5.5388 | 5.5417 | 5.5445 | 5.5476 | 5.5505 |
| 71 | 5.5534 | 5.5563 | 5.5592 | 5.5622 | 5.5651 | 5.5681 | 5.571 | 5.574 | 5.5769 | 5.5799 |
| 72 | 5.5828 | 5.5858 | 5.5888 | 5.5918 | 5.5948 | 5.5978 | 5.6008 | 5.6038 | 5.6068 | 5.6098 |
| 73 | 5.6128 | 5.6158 | 5.6189 | 5.6219 | 5.625 | 5.628 | 5.6311 | 5.6341 | 5.6372 | 5.6403 |
| 74 | 5.6433 | 5.6464 | 5.6495 | 5.6526 | 5.6557 | 5.6588 | 5.662 | 5.5651 | 5.6682 | 5.6713 |
| 75 | 5.6745 | 5.6776 | 5.6808 | 5.684 | 5.6871 | 5.6903 | 5.6935 | 5.6967 | 5.6999 | 5.7031 |
| 76 | 5.7063 | 5.7095 | 5.7128 | 5.716 | 5.7192 | 5.7225 | 5.7257 | 5.729 | 5.7323 | 5.7356 |
| 77 | 5.7388 | 5.7421 | 5.7454 | 5.7488 | 5.7521 | 5.7554 | 5.7588 | 5.7621 | 5.7655 | 5.7688 |
| 78 | 5.7722 | 5.7756 | 5.779 | 5.7824 | 5.7858 | 5.7892 | 5.7926 | 5.7961 | 5.7995 | 5.803 |
| 79 | 5.8064 | 5.8099 | 5.8134 | 5.8169 | 5.8204 | 5.8239 | 5.8274 | 5.831 | 5.8345 | 5.8331 |
| 80 | 5.8416 | 5.8452 | 5.8488 | 5.8524 | 5.856 | 5.8596 | 5.8633 | 5.8669 | 5.8705 | 5.8742 |
| 81 | 5.8779 | 5.8816 | 5.8853 | 5.889 | 5.8927 | 5.8965 | 5.9002 | 5.904 | 5.9078 | 5.9116 |
| 82 | 5.9154 | 5.9192 | 5.923 | 5.9269 | 5.9307 | 5.9346 | 5.9385 | 5.9424 | 5.9463 | 5.9502 |
| 83 | 5.9542 | 5.9581 | 5.9621 | 5.9661 | 5.9701 | 5.9741 | 5.9782 | 5.9822 | 5.9863 | 5.9904 |
| 84 | 5.9945 | 5.9985 | 6.0027 | 6.0069 | 6.011 | 6.0152 | 6.0194 | 6.0237 | 6.0279 | 6.0322 |
| 85 | 6.0364 | 6.0407 | 6.045 | 6.0494 | 6.0537 | 6.0581 | 6.0625 | 6.0669 | 6.0714 | 6.0758 |
| 86 | 6.0803 | 6.0848 | 6.0893 | 6.0929 | 6.0985 | 6.1031 | 6.1077 | 6.1123 | 6.117 | 6.1217 |
| 87 | 6.1264 | 6.1311 | 6.1359 | 6.1407 | 6.1455 | 6.1503 | 6.1552 | 6.1601 | 6.165 | 6.17 |
| 88 | 6.175 | 6.18 | 6.185 | 6.1901 | 6.1952 | 6.2004 | 6.2055 | 6.2107 | 6.216 | 6.2212 |
| 89 | 6.2265 | 6.2319 | 6.2372 | 6.2426 | 6.2431 | 6.2536 | 6.2591 | 6.2646 | 6.2702 | 6.2759 |
| 90 | 6.2816 | 6.2673 | 6.293 | 6.2988 | 6.3047 | 6.3106 | 6.3165 | 6.3225 | 6.3285 | 6.3346 |
| 91 | 6.3408 | 6.3469 | 6.3532 | 6.3595 | 6.3658 | 6.3722 | 6.3787 | 6.3852 | 6.3917 | 6.3984 |
| 92 | 6.5051 | 6.4118 | 6.4187 | 6.4255 | 6.4325 | 6.4395 | 6.4466 | 6.4538 | 6.4611 | 6.4584 |
| 93 | 6.5758 | 6.4833 | 6.4909 | 6.4985 | 6.5063 | 6.5141 | 6.522 | 6.5301 | 6.5328 | 6.5484 |
| 94 | 6.5548 | 6.5632 | 6.5718 | 6.5805 | 6.5893 | 6.5982 | 6.6072 | 6.6164 | 6.6258 | 6.6352 |
| 95 | 6.6449 | 6.6546 | 6.6646 | 6.6747 | 6.6849 | 6.6954 | 6.705 | 6.7169 | 6.7279 | 6.7392 |
| 96 | 6.7507 | 6.7624 | 6.7744 | 6.7866 | 6.7991 | 6.8119 | 6.825 | 6.8384 | 6.8522 | 6.8663 |
| 97 | 6.8808 | 6.8957 | 6.911 | 6.9268 | 6.9431 | 6.96 | 6.9774 | 6.9954 | 7.0141 | 7.0335 |
| 98 | 7.0537 | 7.0749 | 7.0969 | 7.1201 | 7.1444 | 7.1701 | 7.1973 | 7.2262 | 7.2571 | 7.2904 |
| 99 | 7.3263 | 7.3656 | 7.4089 | 7.4573 | 7.5121 | 7.5758 | 7.6521 | 7.7478 | 7.8782 | 8.0902 |
