# Supplementary material for: Evaluation of Laboratory Management Based on a Combination of TOPSIS and RSR Methods: A Study in 7 Provincial Laboratories of China
Source: Front Public Health. 2022 Jul 11;10:883551. doi: 10.3389/fpubh.2022.883551 (PMC9309487; doi:10.3389/fpubh.2022.883551)
Supplement: Supplementary file 3 [file Table_2.docx]

**Appendix A**

See Table A2.

Table A2 Percentile $P_{X}$and the corresponding Probit values at different levels

| Levels | Percentile$P_{X}$ | Probit |
| --- | --- | --- |
| 3 | <P15.866 | <4 |
|  | P15.866～ | 4～ |
|  | P84.134～ | 6～ |
| 4 | <P6.681 | <3.5 |
|  | P6.681～ | 3.5～ |
|  | P50～ | 5～ |
|  | P93.319～ | 6.5～ |
| 5 | <P3.593 | <3.2 |
|  | P3.593～ | 3.2～ |
|  | P27.425～ | 4.4～ |
|  | P72.575～ | 5.6～ |
|  | P96.407～ | 6.8～ |
